# Linking Individual Natural History to Population Outcomes in Tuberculosis

**DOI:** 10.1093/infdis/jix555

**Published:** 2017-11-02

**Authors:** Phillip P Salvatore, Alvaro Proaño, Emily A Kendall, Robert H Gilman, David W Dowdy

**Affiliations:** 1 Department of Molecular Microbiology and Immunology, The Johns Hopkins Bloomberg School of Public Health, Baltimore, Maryland; 2 Laboratorio de Investigación en Enfermedades Infecciosas, Laboratorio de Investigación y Desarrollo, Facultad de Ciencias y Filosofía, Universidad Peruana Cayetano Heredia, Lima, Peru; 3 Division of Infectious Diseases, The Johns Hopkins University School of Medicine, Baltimore, Maryland; 4 Asociación Benéfica PRISMA, Lima, Peru; 5 Department of International Health, Baltimore, Maryland; 6 Department of Epidemiology, The Johns Hopkins Bloomberg School of Public Health, Baltimore, Maryland

**Keywords:** tuberculosis, natural history, disease progression, spontaneous remission, mathematical models

## Abstract

**Background:**

Substantial individual heterogeneity exists in the clinical manifestations and duration of active tuberculosis. We sought to link the individual-level characteristics of tuberculosis disease to observed population-level outcomes.

**Methods:**

We developed an individual-based, stochastic model of tuberculosis disease in a hypothetical cohort of patients with smear-positive tuberculosis. We conceptualized the disease process as consisting of 2 states—progression and recovery—including transitions between the 2. We then used a Bayesian process to calibrate the model to clinical data from the prechemotherapy era, thus identifying the rates of progression and recovery (and probabilities of transition) consistent with observed population-level clinical outcomes.

**Results:**

Observed outcomes are consistent with slow rates of disease progression (median doubling time: 84 days, 95% uncertainty range 62–104) and a low, but nonzero, probability of transition from disease progression to recovery (median 16% per year, 95% uncertainty range 11%–21%). Other individual-level dynamics were less influential in determining observed outcomes.

**Conclusions:**

This simplified model identifies individual-level dynamics—including a long doubling time and low probability of immune recovery—that recapitulate population-level clinical outcomes of untreated tuberculosis patients. This framework may facilitate better understanding of the population-level impact of interventions acting at the individual host level.

## Teaser

Experimental models of tuberculosis are rarely interrogated for consistency with observed clinical outcomes at the population level. We developed a mathematical framework of tuberculosis to describe individual-level rates of disease progression and recovery that are consistent with reported clinical outcomes.

Tuberculosis remains one of the leading causes of death worldwide, with an estimated 23% of the world’s population infected and 1.4 million individuals dying of tuberculosis in 2015 [1, 2]. The spectrum of disease caused by *Mycobacterium tuberculosis* demonstrates marked heterogeneity in terms of pathological presentation [3], incubation period [4], infectiousness [5], treatment responses [6], and other key clinical characteristics. While experimental studies have described underpinning biological mechanisms [7], and epidemiological studies have identified risk factors for tuberculosis progression at the population level [8], integrating these distinct approaches remains a complex task.

Epidemiological models are often utilized to make inferences about dynamics of complex systems, such as transmission of drug-resistant tuberculosis [9] and population-level impacts of various interventions [10]. In many such models, however, individual-level temporal dynamics and pathological processes (such as disease onset, progression, cure, and death) are simplified as population-level rates or probabilities. In contrast, within-host models can help disentangle individual-level dynamics of *M. tuberculosis* replication, host immune cell responses, cytokine signaling, pathology, and bacterial metapopulations [11–15]. Most within-host models of tuberculosis have uncertain applicability to human epidemics, however, as they draw on biological observations of experimental animal infection that have important dissimilarities with key aspects of human disease—including long-term asymptomatic latency, spontaneous self-resolution, and heterogeneity in disease outcome [16]. There is therefore a critical gap in our understanding, namely the linkage of individual-level pathological processes to population-level clinical outcomes. Filling this gap could help to better predict the population-level effects of interventions—from better treatment for drug-resistant tuberculosis to earlier diagnosis and linkage to care—for which individual-level biological effects may be easier to measure.

In this study, we present a mathematical framework to address this knowledge gap using a simplified biological representation of tuberculosis progression across a population of individuals with incipient active tuberculosis. In developing this framework, we aimed to create the simplest possible representation of biological processes that could be compared against observed population-level clinical outcomes. We then calibrate this system to characteristics of the natural history of tuberculosis observed in empirical studies of patients in the prechemotherapy era. The primary objective of this study is to identify individual-level characteristics of tuberculosis disease progression which could—when simulated in a simplified system over large populations of immunocompetent individuals—successfully recapitulate clinical outcomes of untreated tuberculosis at the population level.

## METHODS

### Objective

To better simulate the temporal dynamics and heterogeneous outcomes of disease progression in clinical populations, we developed an individual-based, stochastic mathematical model of pulmonary tuberculosis progression in the human host. To link this model to population-level clinical outcomes, we drew upon data from epidemiological studies describing the natural history of tuberculosis before the worldwide introduction of modern antimycobacterial therapy (or the emergence of the HIV/AIDS pandemic) [17]. A systematic review of these studies estimated that, among adults diagnosed with sputum smear-positive tuberculosis, the average duration of disease was 3 years, 55% would die within 5 years, and 28% would eventually spontaneously resolve without chemotherapy [17]. We therefore sought to ascertain the individual-level characteristics of tuberculosis progression and resolution that could replicate similar clinical outcomes in large simulated populations.

### Conceptual Framework

**Figure 1.**
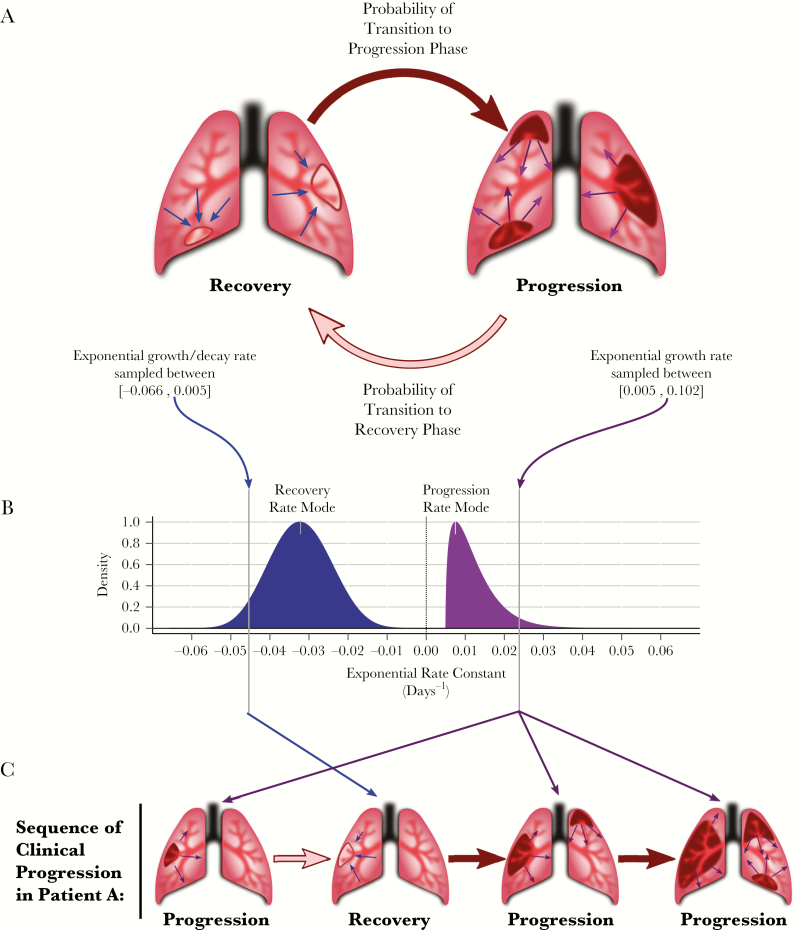
Individual-level model framework of progression and recovery in tuberculosis. *A*, Each patient’s disease is modeled through time as a sequence of transitions between disease progression and disease recovery. *B*, A patient may take her rate of progression and her rate of recovery from a range of plausible rates, represented by probability densities across possible values of progression (black density) and recovery (gray density). The shape of these densities is determined by the specific value of the rate mode parameters. Within each cohort, a value from each of these densities (depicted by vertical lines in the plot) is stochastically sampled to characterize each patient’s infection. *C*, In the case of an arbitrary simulated Patient A, disease development begins in the progression phase, during which growth is characterized by the patient’s sampled rate of progression. At any time (with a given weekly probability), the infection may transition to recovery, during which growth/decay is characterized by the patient’s sampled rate of recovery. Similarly, at any subsequent time, the infection may transition from recovery to the progression phase, with the same rate of progression as sampled previously. The concomitant changes in Patient A’s disease burden are represented in Figure 2.

To construct a conceptual framework to address the primary objective, we made the simplifying assumption that, once infected, individuals exist in 1 of 2 clinical phases: disease progression or stabilization/recovery (Figure 1A). During these phases, the disease burden may increase and symptoms worsen (progression), or the disease burden may stabilize and symptoms either improve or worsen only slowly (recovery). To capture the myriad host and pathogen modifiers that influence an individual’s disease phenotype [18–20], rates of progression and recovery are modeled at the individual level to allow for variability from one individual to the next (see rate distributions in Figure 1B). Each individual’s course of disease may then be simulated as a rate of progression, a rate of recovery, and a set of Markov probabilities that define the transitions between these 2 phases (see Figure 1C).

While these conceptual phases of progression and recovery are simplifications of the complex pathophysiology of tuberculosis infection [21], they are analogous to experimentally observable dynamics of bacterial replication and immune responses in vivo [7]. Unlike biological within-host models of tuberculosis [11–14, 22], this model does not attempt to capture the complex and diverse immunological and pathophysiological mechanisms that influence clinical outcomes in any given individual. Rather, for simplicity and ease of understanding, we use “disease burden” as a mathematical benchmark that is likely associated with clinical outcomes (eg, individuals who develop a higher burden may experience more severe symptoms, increased risk of mortality, and other pathological characteristics such as increased infectiousness) [18, 23, 24]. In this framework, “disease burden” should not be interpreted as a direct representation of bacillary load; instead, disease burden in this model represents a composite measure of characteristics of tuberculosis disease—such as pulmonary pathology, cough frequency, immunological exhaustion, chronic weight loss, etc., in addition to bacillary load—that correlate with the progression of clinical disease in patients. In this context, the simulated disease burden is not a verifiable quantity per se, but rather an instrument to relate the observable rates of progression and recovery in human populations to potentially measurable analogues of bacillary growth and decline in experimental systems.

We next define a conceptual “symptom threshold” as the disease burden above which active tuberculosis becomes symptomatic and clinically recognizable (ie, as would be observable in prechemotherapy studies of smear-positive tuberculosis patients) [17]. Inversely, an infection in which the disease burden falls below this threshold represents an apparent self-resolution of symptomatic tuberculosis. Similarly, we define a “death threshold” —another conceptual construct—as the disease burden beyond which death would occur. (Similar techniques have been used to define “detectability” and “life-threatening” thresholds in models of cancer progression [25].) Using disease burden as a reference frame for clinical characteristics thus allows the duration and clinical status of each simulated case to be tracked.

### Individual-Level Dynamics

Each patient is assumed to start at a disease burden of 1 (arbitrary) unit in the progression phase. The start of each simulation therefore represents the time at which a pathophysiological process toward progression to symptomatic, active disease (ie, “incipient” tuberculosis) begins. Therefore, each patient is considered clinically silent and epidemiologically undetectable in our analyses until the patient’s disease burden exceeds the symptom threshold for the first time. In calculating the disease burden at each discrete time step, we assume that progression and recovery follow the properties of a simple exponential process, with a single rate constant describing net growth (or decay) over time for a given individual in a given phase. We assume that the range of plausible growth rates during the progression phase (Figure 1B, black distribution) is higher than the range of plausible growth (or decay) rates during the recovery phase (Figure 1B, gray distribution). While the disease progression of 2 simulated patients may exhibit different exponential growth and decay rates, the simulated disease within each individual host is assigned a single representative rate for the progression phase and a single representative rate for the recovery phase, sampled from the plausible ranges of each distribution (illustrated as the vertical lines on each curve in Figure 1B).

**Figure 2.**
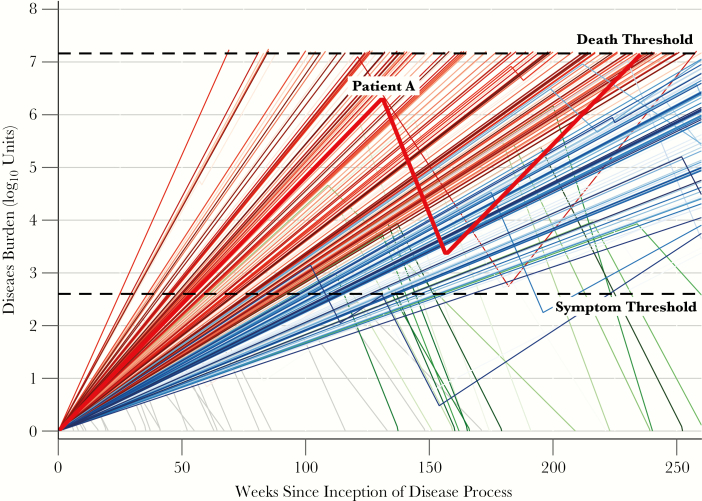
Disease burdens of a simulated population over time. Patients whose infections exceed the “symptom threshold” (a mathematical benchmark) are tracked until death (when the bacillary burden exceeds a mathematical “death threshold”, colored in red) or self-resolution (when the burden falls below the “symptom threshold”, colored in green). Patients who continue to experience active tuberculosis (ie, without exceeding the death threshold or experiencing self-resolution) after 5 years are colored in blue. Patients who never develop symptomatic disease (ie, never surpass the symptom threshold) are plotted in grey. All disease burdens depicted were generated using the same model parameters and represent the population variability in progression/recovery rates as well as stochastic transitions between phases of infection. The disease burden of an arbitrary Patient A quantitatively depicts the progression of disease diagrammed graphically in Figure 1C; note that the rate (slope) of progression for Patient A is the same throughout her life (ie, both before and after recovery).

As a simulated course of disease progresses, each individual may transition between progression and recovery phases (illustrated for an arbitrary simulated “Patient A” in Figure 1C). Mathematically, these transitions occur probabilistically, independent of disease burden or history, and correspond to switches from the progression rate to the recovery rate, or vice versa (black and gray arrows in Figure 1B and 1C). If, at any point, the disease burden of a symptomatic patient exceeds the “death threshold”, the patient is classified as having died of tuberculosis. Conversely, a patient whose burden declines below the “symptom threshold” is classified as an apparently self-resolved case; these cases may relapse with symptomatic tuberculosis during the 5 years of simulation (see “Patient A” in Figure 2). Patients whose disease burden declines below the starting value of 1 unit are classified as cured, with no further possibility of disease progression. Thus, for each patient the duration of disease can be calculated as the continuous time spent with a disease burden between the “symptom threshold” and the “death threshold.” A cohort of simulated patients is then assembled to estimate population-level clinical characteristics such as tuberculosis mortality, spontaneous resolution, and disease duration, accounting for variation in progression/recovery rates from one patient to the next as well as stochastic transition events from progression to recovery and back. A representative cohort of 250 simulated patient trajectories (representing a single set of population-level parameter distributions) is illustrated in Figure 2; emphasized is the infection trajectory of “Patient A” (diagramed qualitatively in Figure 1C).

### Analytic Methods

To evaluate the likely values of model parameters (progression/recovery rates and phase transition probabilities), we implemented a Bayesian sampling-importance-resampling algorithm [26]. In this approach, a range of reasonable (“prior”) values was defined for each model parameter (Table 1). These prior ranges were taken as uniform distributions, on either the logarithmic- or log-modulus-transformed scales [27], bounded as shown in Table 1. Latin hypercube sampling [28] was then utilized to randomly draw 2 million sets of parameter values; each set was subsequently used to simulate a population of 1000 patients with untreated tuberculosis using the drawn values for the 2 transition probabilities to inform stochastic realizations of the sequence of progression and recovery in each individual patient. The drawn values of the progression rate and recovery rate for the cohort represented the cohort’s population modal progression and recovery rate, respectively, with each individual’s rates drawn from a beta distribution (chosen to provide central tendency within defined upper and lower bounds) around each mode. Beta distributions were parameterized by the modal value and a concentration parameter of κ = 20 to maintain a clear central tendency in each population. Individuals who never reached the symptom threshold over 5 years were dropped from the analysis. All other individuals were simulated until death (reaching the death threshold, colored in red in Figure 2), spontaneous recovery (again falling below the symptom threshold, colored in green in Figure 2), or 5 years of symptomatic disease (colored in blue in Figure 2) —reflecting the 5-year mortality/recovery data to which our model was calibrated [17]. Outcomes in each cohort were aggregated to calculate the population-level simulated outcomes described below.

**Table 1.** Parameter Values Used to Define Upper and Lower Bounds of Sampling Ranges

Parameter	Sampled Range	References	Notes
Death threshold (log_10_ units)	7.0–10.0^a^	[37–39]	Burdens in animal models rarely measure greater than 10^9^ units per lung.
Width of the window between symptom and death thresholds (log_10_ units)^b^	4.0–7.0^a^	[39–41]	Burdens in animals with asymptomatic infection may be as high as 10^3^ units.
Progression → Recovery transition rate	0.001–0.35 per week^a,c^	Derived	Assume 5% transition per year, and no more than 75% transition per month.
Recovery → Progression transition rate	10^−5^–10^−4^ per week^a,d^	[42]	Probability of reactivation in latent infections estimated to be 0.8% per year.
Mode of the progression phase growth rate	0.035–0.714 per week^e,f^	[39–41]	Assume patients progress from onset to death in 3–60 months, assuming 10^4^ unit symptom window.
Mode of the recovery phase growth rate	(−0.462)−0.035 per week^e,g^	[43]	Assume patients self-resolve at 1/4 the rate of chemotherapeutic recovery (as defined by time to sputum conversion).

^a^Range sampled uniformly on the log_10_ scale.

^**b**^The symptom threshold in a given simulation is derived by value of the death threshold and the value of the symptom window width.

^c^A 0.35 weekly rate of transition is equivalent to a 75% monthly probability of transition.

^d^Rates in this range are equivalent to 0.05%–5.0% probabilities of transition.

^e^Range sampled uniformly on the log-modulus scale.

^f^Progression rates in this range are equivalent to net population doubling times in the range of 6–137 days.

^g^Progression rates in this range are equivalent to the range of a population half-life of 11 days to a population doubling time of 137 days.

To identify those parameter values most consistent with observed population-level data, we assigned each cohort a pseudolikelihood, defined as the joint probability density of the simulated cohort’s population-level characteristics according to estimated density functions for 3 key summary statistics of observed prechemotherapy era cohorts: 55% case-fatality ratio within 5 years of symptom onset, median symptom duration of 3 years, and spontaneous resolution of ≥10% of cases [17]. (For further details, see Importance Resampling in the Supplementary Methods.) After assigning a pseudolikelihood to each simulated cohort, we resampled 2 million cohorts, with replacement, proportional to the pseudolikelihood [26]. The resampled (posterior) distribution therefore represents—in weighted fashion—those cohorts (and their corresponding parameter values) with the best fit to historical data. We define 95% uncertainty ranges (UR) as the 2.5th and 97.5th percentiles of each parameter’s value, across the posterior distribution.

A multivariate sensitivity analysis was performed by computing the partial rank correlation coefficient (PRCC) between each of the 6 input parameters (2 transition probabilities, modal progression rate, modal recovery rate, symptom threshold, and death threshold) and the pseudolikelihood of each plausible cohort. This analysis identifies those parameters that most strongly influence the ability of the simulated data to fit the observed data, adjusting for all other parameters simultaneously. Based on the results of this sensitivity analysis, an additional post-hoc nonparametric Spearman correlation was tested between the probability of transitioning from progression to recovery and the modal progression rate.

Mathematical formulae, prior distributions, likelihood functions, importance resampling, and further technical details are provided in the Supplementary Methods. All statistical computing was performed using R version 3.2.2 (R Foundation for Statistical Computing, Vienna, Austria).

## RESULTS

**Figure 3.**
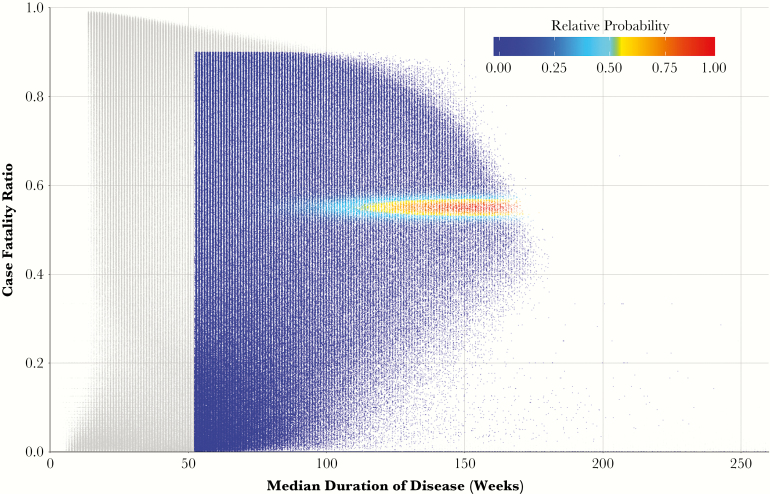
Weighting process of 2 million simulated cohorts, according to fit with observed clinical data. Each point represents the results of a cohort of 1000 simulated patients, plotted according to the 5-year case-fatality ratio and median duration of disease amongst symptomatic patients who die or self-resolve. Each point is colored according to its weight (probability of being resampled to generate the final, or posterior, distribution), measured from 0 to 1 (the maximum likelihood of cohort results). Points colored in grey represent those cohorts with joint likelihoods equal to zero. (Not depicted are the 5% of simulations in which no patients developed symptoms.)

Of the 2 million simulated patient cohorts, 551100 (27%) had results deemed consistent with historical estimates of tuberculosis natural (ie, nonzero pseudolikelihoods, see the Supplementary Results for further details). Figure 3 presents the case-fatality ratios and median durations of disease of all simulated cohorts; the 551100 plausible cohorts are colored according to the pseudolikelihood of each. After weighting (resampling) cohorts according to these pseudolikelihoods, the median case-fatality ratio was 55% (interquartile range [IQR]: 54%, 56%), the median duration of disease for the 50th percentile of cohorts was 2.5 years (IQR: 2.1, 2.8), and the median proportion of self-resolving cases was 28% (IQR: 19%, 37%), consistent with empirical calibration targets (55% case fatality, mean 3-year symptom duration, 28% self-resolved over 10 years) [17].

**Figure 4.**
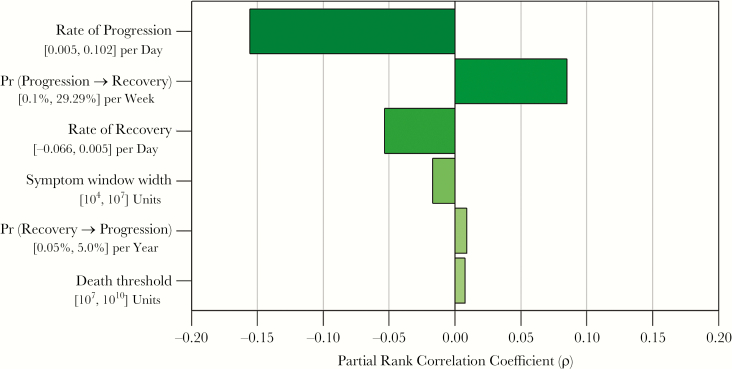
Association between key model parameters and population-level clinical results. Each bar represents the partial rank correlation coefficient of the association between each model parameter and the joint likelihood of cohort results (ie, how closely each cohort fits the observed data). Beneath each parameter label is the sampling range from which parameter values were sampled.

**Figure 5.**
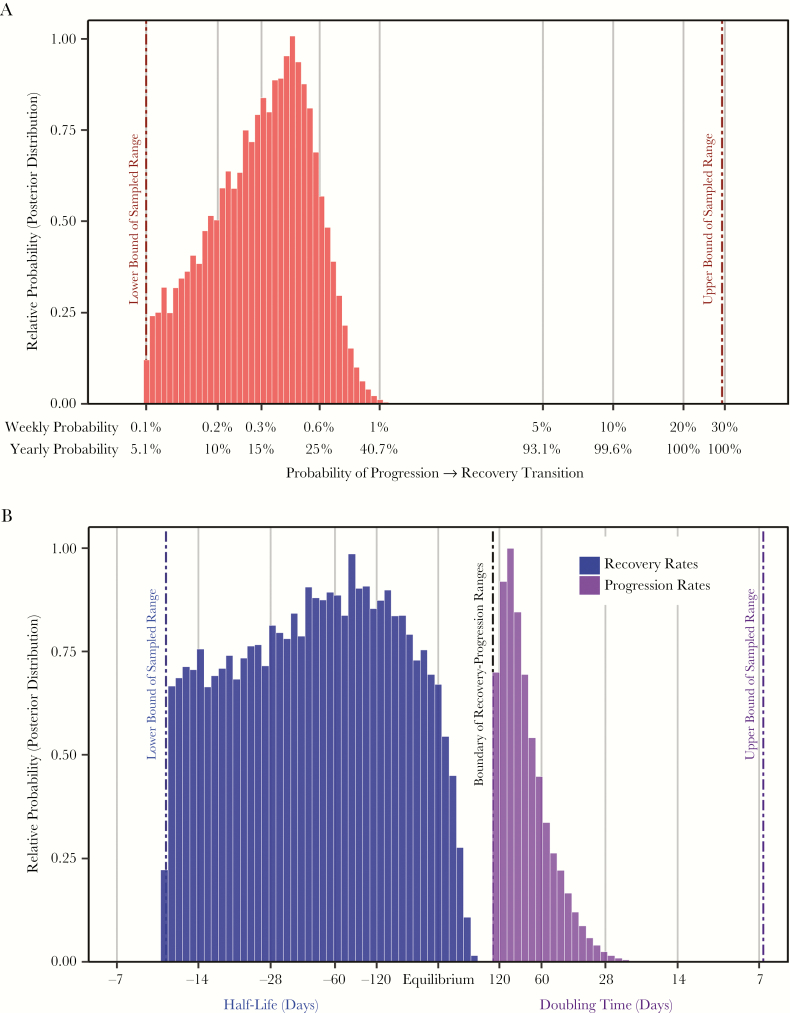
Values of key parameters consistent with observed cohort data. Model input values (prior distributions) were sampled uniformly on log-transformed or log-modulus-transformed scales between reasonable bounds selected on an a priori basis (denoted by vertical dashed lines). Histogram densities show the proportion of 2 million weighted parameter values that were most consistent with observed cohort data from the prechemotherapy era (posterior distributions). *A*, Distributions of probabilities of transition from progression phase to recovery phase on weekly and annualized scales. Simulations most consistent with observed data therefore primarily contain transition probabilities in the lower half of the initially sampled range (ie, left-hand portion of the graph). The median transition probability among these data-consistent simulations (16% per year) implies that 50% of patients transition within 4.2 years (0.5 = *e*^−0.16 × 4.2^), provided they survive that long. *B*, Distributions of progression rates and recovery rates during the progression phase (gray) and the recovery phase (black). In this graph, a progression/recovery rate of zero implies no change in disease burden with time. Recovery rates less than zero are therefore also displayed as half-lives (ie, duration of time required for the disease burden to be cut in half), while progression rates are displayed as doubling times (ie, duration of time required for the disease burden to double).

The correlation between each input parameter value and the fit between simulated and observed data is presented in Figure 4. The probability of transition from the progression phase to the recovery was also strongly correlated with model fit, and plausible models are consistent with a median yearly transition probability of 16% (95% UR: 11%, 21%; Figure 5A) The most important determinant of model fit was the rate of disease progression, and plausible results indicate a median progression rate of 0.0083 per day (95% UR: 0.0066, 0.011), equivalent to an exponential doubling time of 84 days (95% UR: 62, 104; Figure 5B, black posterior). This range can be interpreted as the doubling times of tuberculosis “disease burden” that are consistent with observed data on case-fatality, duration of clinical disease, and probability of self-resolution [17]. The rate of disease progression and the probability of transition from progression to recovery were correlated among plausible cohorts (Spearman’s ρ = .68, *P* < .01).

The association between the rate of recovery and model fit to observed data was less strong (Figure 4). Model results suggest a median recovery rate of −0.014 per day (95% UR: −0.032, −0.0052), equivalent to an exponential half-life of 48 days (95% UR: 22, 133; Figure 5B, gray posterior). Sensitivity analysis indicated that model fit was not associated with the value of the symptom threshold, death threshold, or probability of transition from recovery to progression (|PRCC|<0.02 for each; see also Supplementary Figures S1–S3).

To illustrate the potential application of this framework for investigating the impacts of individual-level interventions, we simulated cohorts with various diagnostic strategies (detailed further in the Supplementary Methods). In this example, our model was able to recapitulate global estimates of case fatality in the presence of partial diagnosis and treatment coverage (17.3%) [2] and illustrate how those gains in mortality could be achieved without observing substantial reductions in incidence (see the Supplementary Results for further details).

## DISCUSSION

This study sought to identify individual-level tuberculosis disease characteristics that were consistent with historical population-level clinical outcomes, using a simplified model. As our model is calibrated to clinical characteristics of symptomatic tuberculosis, it should be interpreted as representative of incipient and active tuberculosis (but not long-term latency). The simulated case-fatality (median 55%), duration of disease (median 129 weeks), and proportion of self-resolved cases (median 28%) indicate that this simplified structure can capture the basic dynamics of clinical tuberculosis progression in human populations. Our primary results suggest that, under physiological conditions in realistic populations, active tuberculosis may reasonably be represented as a slow rate of disease progression (median 84-day doubling time) and a low probability of transitioning from progression to recovery (16% per year).

This modeling approach deliberately utilized a minimal parameterization of complex disease processes, but many possible complex models may also be consistent with observed clinical characteristics of tuberculosis progression. Therefore, the results presented here must be interpreted in the context of this framework and the analytical approach utilized.

It may be useful to provide intuitive context for these results. For example, we fit our model to a review of prechemotherapy era studies suggesting that tuberculosis patients experienced symptoms for an average of 3 years before death or symptom resolution [17]. Our estimated median doubling time of 84 days (Figure 5B, black posterior) would generate a net 10^4^ unit increase in disease burden (the minimum difference between the “symptom threshold” and “death threshold”) in 3.1 years of continuous progression. Our estimated 16% yearly probability—equivalent to a 29% probability of transition in the first 2 years—likewise reflects the empirical estimate (to which our data were fit) that 28% of untreated tuberculosis patients experience spontaneous resolution [17].

The data from this model also offer useful context with which to view experimental results from in vitro and in vivo models. For example, murine data suggest much faster disease progression (physiological doubling times of 2–3 days [29, 30]) and shorter duration of disease (median durations of 31 weeks [31]) compared to our results (doubling times of 84 days and median disease duration of 2.5 years). Additionally, our results indicate that most patients who transition from progression to recovery experience sustained reductions in disease burden whereas bacillary burden in murine models may eventually plateau [29, 30] but never declines. This discrepancy illustrates some of the implicit limitations of murine models in the study of human tuberculosis: without treatment, all mice eventually die from tuberculosis whereas many human patients may naturally self-resolve [17]. Our simulation framework—with parameter values calibrated to clinical data in human populations—thus provides an important complement to data from animal models.

A primary limitation of this model framework is its simplification of the complex internal pathophysiological mechanisms of host-pathogen interactions. For example, changes in disease burden are simplified as generalized exponential growth and decay, and transitions between progression and recovery are represented as stochastic processes depending only on the current phase of disease. These simplifications necessarily limit the ability to draw precise mechanistic inference; however, they also allow for simulation of a conceptually tractable measure (disease burden), thereby quantitatively linking individual-level data on disease progression and recovery with observable population-level clinical outcomes. Similarly, we also use mathematical constructs of symptom and death thresholds that have no direct physiological meaning. Importantly, these constructs were not significant determinants of our primary outcome (see Figure 4 and Supplementary Figures S1–S3). Our model is also not capable of calculating the single (“identifiable”) parameter values that are most likely to result in the observed clinical data to which our model was fit. Rather, we sampled from a priori defined ranges and evaluated multiple sets of parameter values that might be consistent. Finally, our calibration strategy used data from historical studies with distinct demographic and epidemiological characteristics; while use of data-driven evidence is an advantage of the methodology, differences in these characteristics between historical and modern populations may limit the generalizability of these results in the contemporary epidemiology of tuberculosis (eg, including HIV, diabetes, and changing age structures).

The development of a model linking individual-level and population-level outcomes opens a variety of avenues for future research and may also help to ground predictions of the population-level impacts of interventions which hinge on the temporal dynamics of individual-level tuberculosis outcomes. For example, the impact of scale-up of screening and diagnostic interventions may be heavily influenced by the distribution of individuals detectable by such interventions, as demonstrated in our simulation of diagnostic and treatment interventions. When patients with high simulated disease burdens are more likely to be diagnosed and treated than patients with low disease burdens, our model accurately reproduced 2015 global estimates of case fatality with high precision, without predicting a major reduction in transmission (see the Supplementary Results). Linking clinical data with this mathematical framework may also be relevant to the transmission of tuberculosis, where a small number of patients (with prolonged symptoms and/or large disease burdens) may generate a majority of new infections [32]. For example, our simulation of diagnosis and treatment implied passive clinical interventions alone may not significantly reduce disease morbidity (as measured by disease burden over time), which may correlate with infectious potential in a population. Identifying such patients with measurable correlates of “disease burden”—such as cough frequency, cavitary lesions, sputum grade, aerosol dispersion, and time to positivity of cultures [33–35]—may augment the impact of diagnostic and treatment interventions on transmission, an effect our model may be able to quantitatively characterize. Finally, this framework holds potential for application to other infections that can be conceptualized as a sequence of transitions between states of varying pathogenesis [36].

In summary, this novel model linking individual-level and population-level outcomes suggests a range of parameters related to tuberculosis progression and recovery that might be consistent with observed clinical outcomes. Among these, we estimate the doubling time of disease burden as 84 days during the progression phase, a half-life of 47 days during recovery, and a probability of transition to recovery of 16% per year. Thus, in human populations, tuberculosis disease burden is likely to grow at a very slow rate, with relatively low probability of switching from progression to recovery in the absence of intervention. While limited by pathological and mechanistic simplifications, this model links within-host and population-level processes to better understand the complex interactions that influence human pathology and disease.

## Supplementary Data

Supplementary materials are available at *The Journal of Infectious Diseases* online. Consisting of data provided by the authors to benefit the reader, the posted materials are not copyedited and are the sole responsibility of the authors, so questions or comments should be addressed to the corresponding author.

## Supplementary Material

PSalvatore FigS1

PSalvatore FigS2

PSalvatore FigS3

PSalvatore FigS4

Supplementary Methods

## Notes

***Financial support*.** This work was supported by the Johns Hopkins Bloomberg School of Public Health (Henger, Cort, Root Scholarship to P. P. S.) and the National Institute of Allergy and Infectious Diseases (3T32AI007417-21A1S1 to P. P. S.; K08AI127908 to E. A. K.; and R21AI094143 to R. H. G.). Additional support was provided by the Bill and Melinda Gates Foundation (contract 23363/Work Order 10 to D. W. D).

***Potential conflicts of interest.*** All authors: No reported conflicts of interest. All authors have submitted the ICMJE Form for Disclosure of Potential Conflicts of Interest. Conflicts that the editors consider relevant to the content of the manuscript have been disclosed.
