# Supplementary figures and images for: Linking Individual Natural History to Population Outcomes in Tuberculosis
Source: J Infect Dis. 2017 Nov 2;217(1):112–21. doi: 10.1093/infdis/jix555 (PMC5853266; doi:10.1093/infdis/jix555)

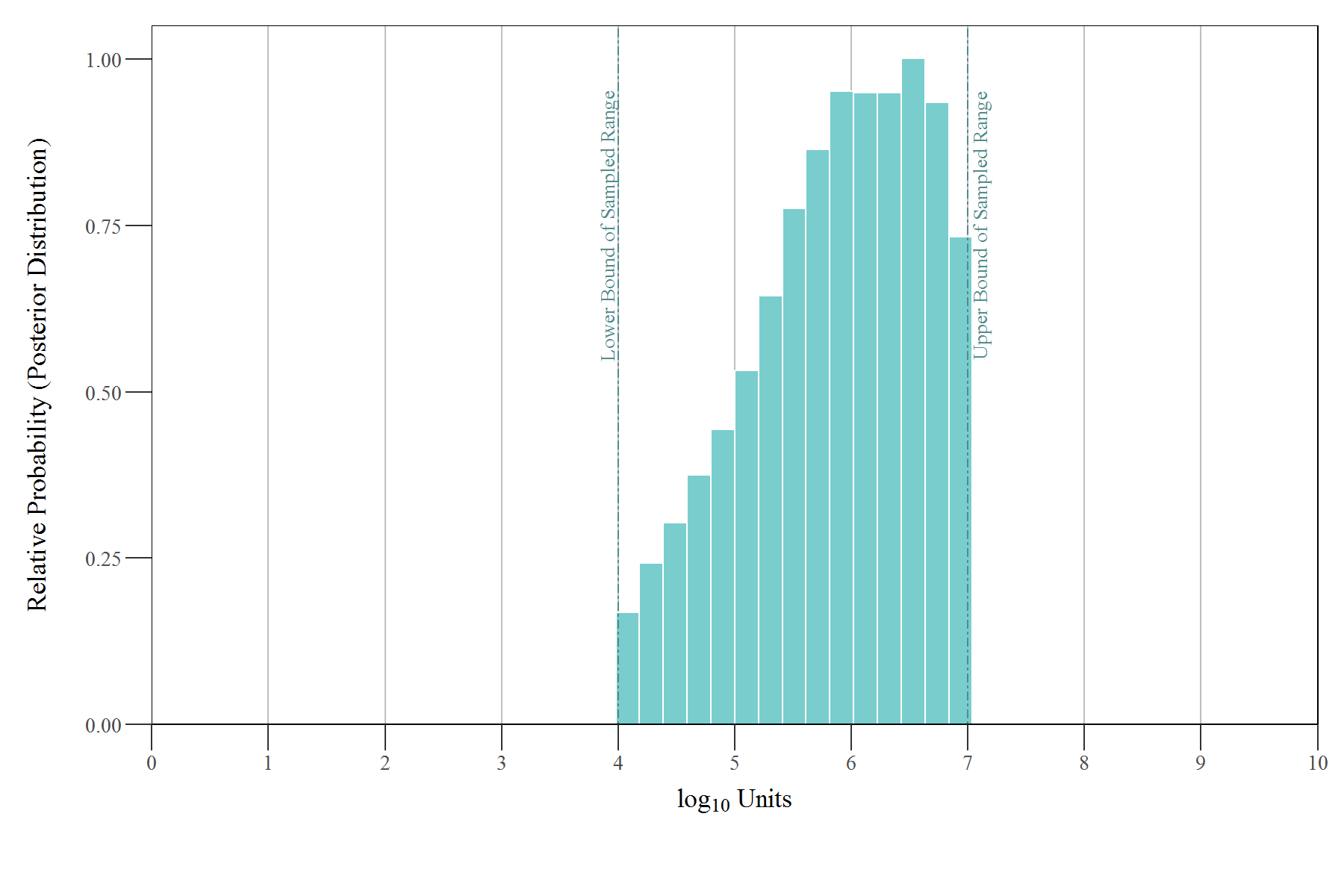

Supplement: PSalvatore FigS1 [file jix555_suppl_supplementary_psalvatore_figs1.png]

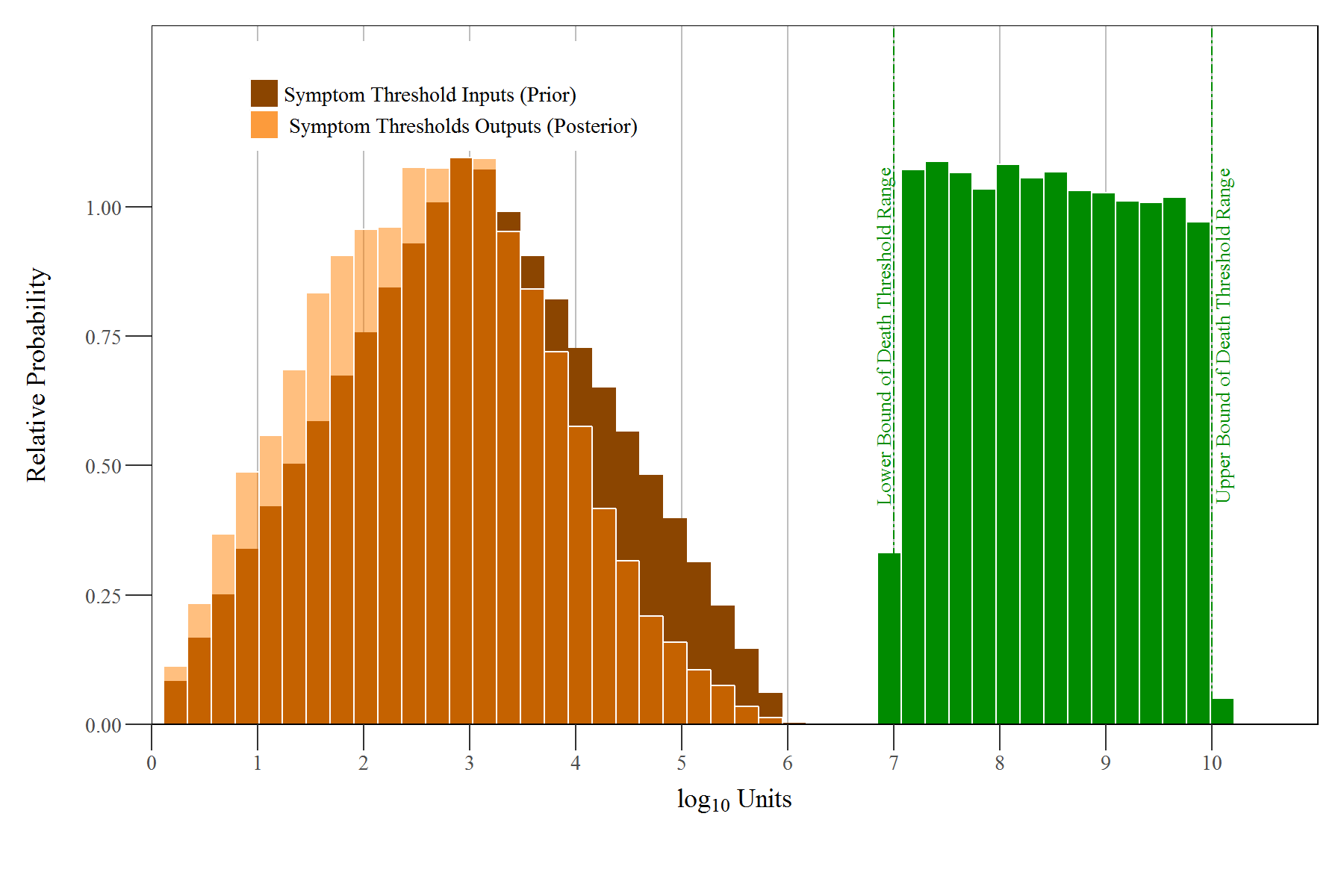

Supplement: PSalvatore FigS2 [file jix555_suppl_supplementary_psalvatore_figs2.png]

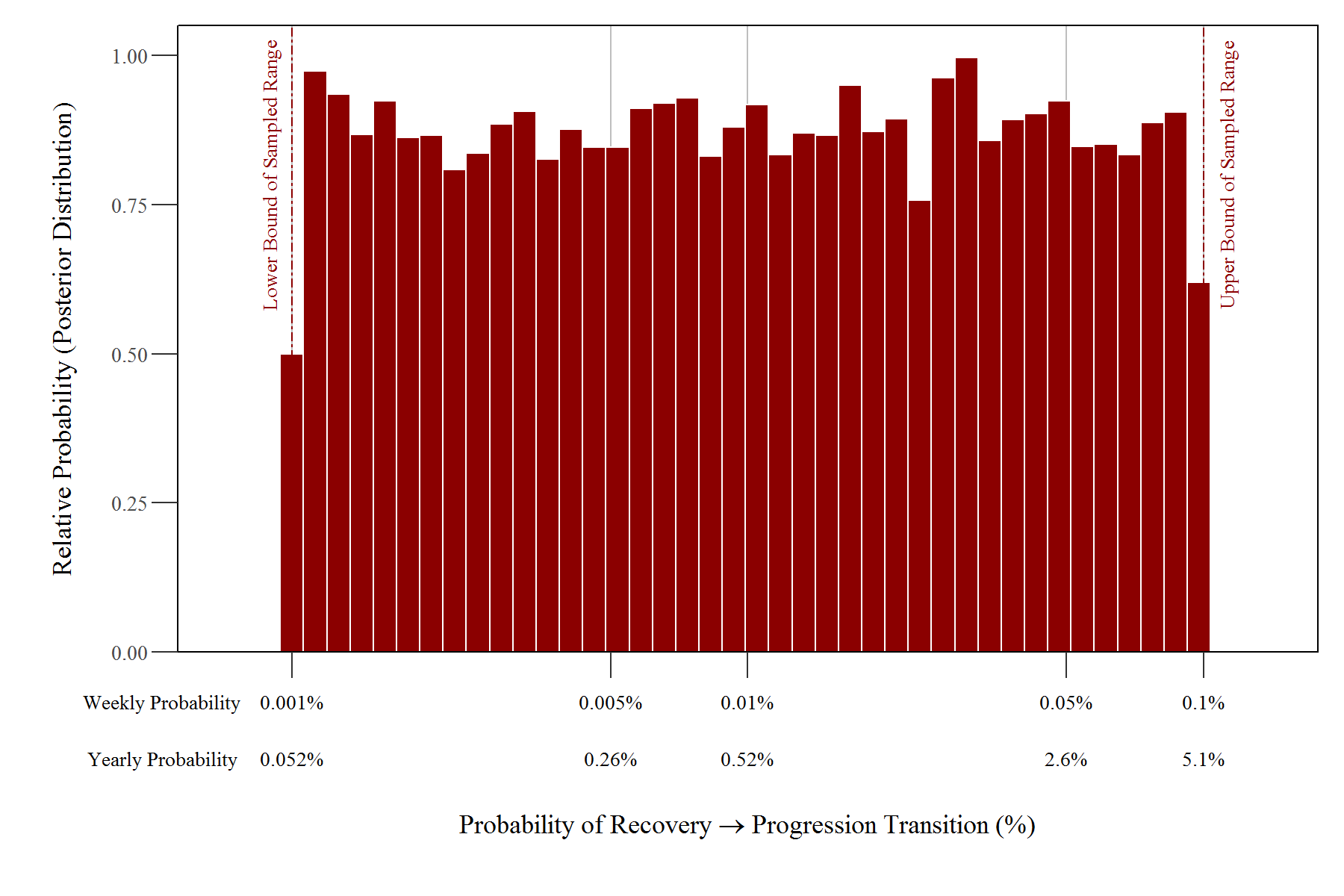

Supplement: PSalvatore FigS3 [file jix555_suppl_supplementary_psalvatore_figs3.png]

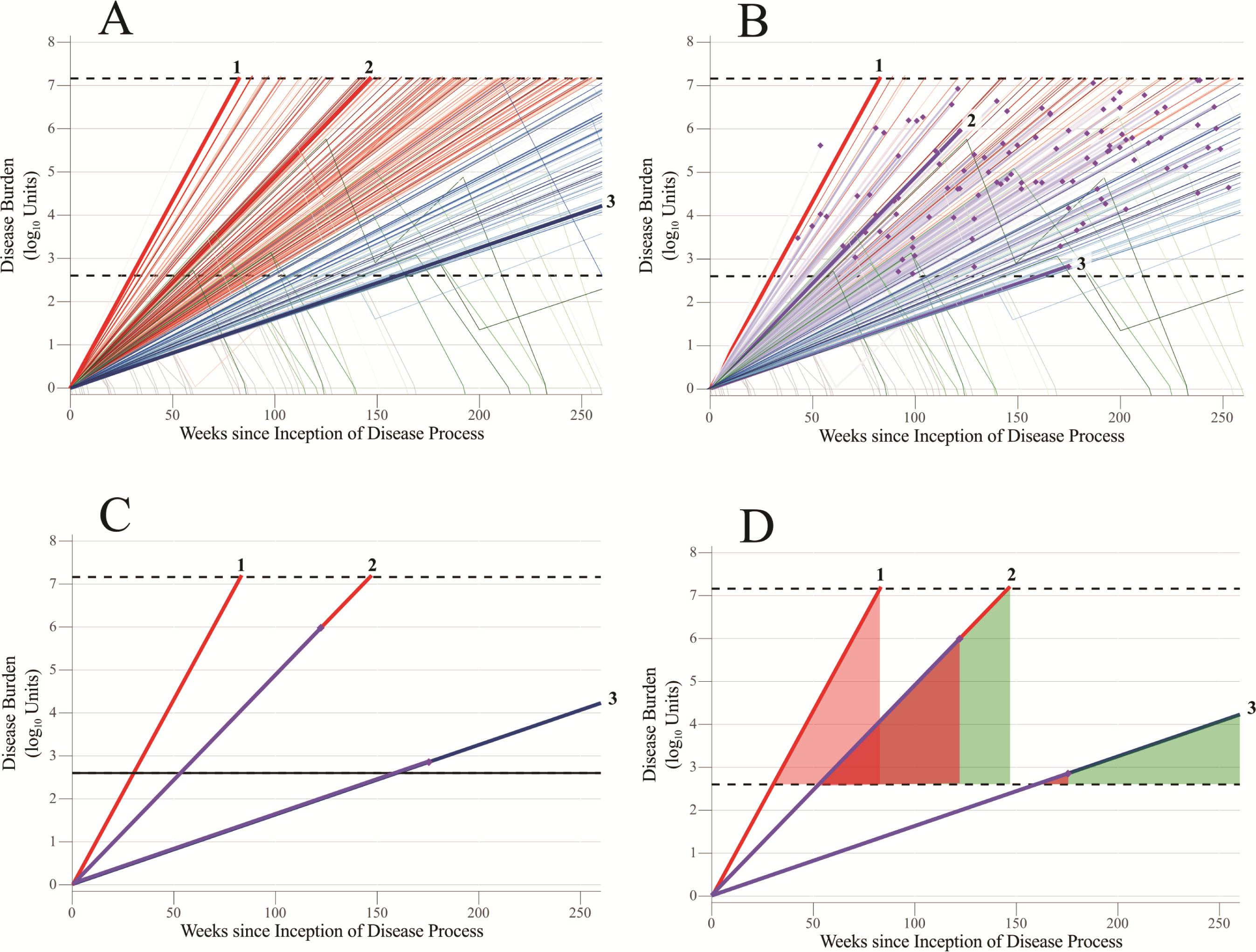

Supplement: PSalvatore FigS4 [file jix555_suppl_supplementary_psalvatore_figs4.png]
