# Supplementary material for: Linking Individual Natural History to Population Outcomes in Tuberculosis
Source: J Infect Dis. 2017 Nov 2;217(1):112–21. doi: 10.1093/infdis/jix555 (PMC5853266; doi:10.1093/infdis/jix555)
Supplement: Supplementary Methods [file jix555_suppl_supplementary_methods.docx]

**Linking individual natural history to population outcomes in tuberculosis**

Phillip P. Salvatore, Alvaro Proaño, Emily A. Kendall, Robert H. Gilman, David W. Dowdy

**Supplementary Content**

**SUPPLEMENTARY METHODS**

This modeling strategy utilizes an agent-based, stochastic approach to simulate the clinical progression of symptomatic TB in individual patients over a course of infection. The clinical progression of disease is parameterized as the simulated disease burden of the infected patient. The individual-level dynamics through which this model functions are conceived as the consequence of two components: a sequence of states of infection, and the progression or recovery of disease during each element of the sequence.

**The Sequence of Infection States**

In clinical populations, a qualitative dichotomy is often observed between TB patients who are experiencing decompensation and concomitant worsening of symptoms and prognosis versus patients who are experiencing improvement in symptom resolution and prognosis whether due to successful treatment or spontaneous self-resolution [1]. Additionally, individual patients may experience one or both of these trends over the course of infection. While these general trends are the manifestations of complex immunological and pathophysiological processes, the trends themselves hold important prognostic information about the likely outcome of the patient.

To quantitatively capture these prognostic qualitative phases, a simplifying assumption is made that, at each discrete time step during the course of a single infection, the disease burden of a patient exists in one of two mutually exclusive states $S^{(t)}\in\left\{ progression , recovery \right\}$. Between any two time steps $\left\{ S^{(t)},S^{(t+1)} \right\}$ , the infection can freely transition between these states. The probability of transition in any time step is dependent only on the current state of infection:

$$S^{(t)}, S^{(t+1)} \sim i.i.d. Pr\left( S^{\left( t+1 \right)} | S^{\left( t \right)} \right)$$

Therefore, the sequence of states that composes any individual infection $\left\{ S^{(0)},{\cdots,S}^{(n)} \right\}$ has the properties of a simple Markov Chain.

**Infection Growth and Decay**

One of the most significant prognostic determinants of TB mortality is pulmonary bacillary burden [2–4]. While experimentally unfeasible to measure in TB patients, bacillary burden is commonly measured in time course studies of infection in animals. Empirical data of changes in bacillary burden has been successfully modeled according to exponential dynamics of bacterial populations, consistent with the underlying biology of bacterial replication [5,6]. Therefore, this within-host model assumes that patient “disease burden” is analogous to, though an indirect instrument of, bacillary burden size.

During any single time step $\Delta t$, the disease burden of a patient is assumed to follow the properties of a simple exponentially reproducing population: $N_{t+1}=N_{t}e^{r_{S^{(t)}}\Delta t}$ where *N_t_* represents the disease burden at time *t* and $r_{S^{(t)}}$represents the exponential rate constant during the interval $\Delta t=[t,t+1)$ in which the infection exists in state $S^{(t)}$. Therefore, when $r_{S^{(t)}}>0$, the disease burden is increasing exponentially; when $r_{S^{(t)}}<0$, the disease burden is decaying exponentially. When $r_{S^{(t)}}=0$, the disease burden is stationary during the interval $\Delta t$ and $N_{t+1}=N_{t}$. The cumulative dynamics of the bacillary burden of any infection can then be summarized as

$${N_{n}=N}_{0}\prod_{t=1}^{n} e^{r_{S^{(t)}}\Delta t} for S=\left\{ S^{(0)},\cdots, S^{(n)} \right\}$$

**Between-Host Variability**

It is notable that, in clinical populations, certain patients are particularly vulnerable to progression once infected while other patients are particularly resilient. This between-host heterogeneity may result from the specific interactions between each human host, the quality of his or her immune response, and the genetic and epigenetic characteristics of the inciting strain/genotype of *M. tuberculosis* [3,7,8]. While recognizing the significant between-host heterogeneity that exists in a population of TB patients, the population as a whole exhibits characteristic trends in disease progression and resolution. For example, relatively few patients progress from symptom onset to death in less than 10 weeks or more than 10 years; the average duration of disease in TB patients has been estimated at 3 years [9]. These population-level characteristics can be used to inform the likely amount of between-host variability in the population.

This between-host variability is captured mathematically as the distribution of exponential rate constants of infections in the population. For each possible state $S^{(t)}\in\left\{ progression , recovery \right\}$, the corresponding rate constant $r_{S^{(t)}}$ is modeled as a beta distribution with parameters $\beta\left( \omega_{S^{(t)}}, \kappa=20 \right)$ transformed over the interval $\left[ \alpha_{S^{(t)}},\zeta_{S^{(t)}} \right]$ where $\omega_{S^{(t)}}$ represents the mode of exponential rate constants in the population of infections in state $S^{(t)}$ , $\kappa$ represents the beta distribution concentration parameter, $\alpha_{S^{(t)}}$ represents the minimum allowable exponential rate constant in state $S^{(t)}$ , and $\zeta_{S^{(t)}}$ represents the maximum allowable exponential rate constant in state $S^{(t)}$. This concentration value was chosen to maintain a clear central tendency in any simulated population, with a standard deviation less than 11% the sampling range ($\max\left( \sigma_{\beta} \right)=\sqrt{\frac{\left( 0.5\kappa\right)^{2}}{\kappa\left( \kappa+1 \right)}}$ ). For any individual infection in the population, $r_{progression}\sim i.i.d. \beta\left( \omega_{progression}, \kappa=20,\alpha_{progression},\zeta_{progression} \right)$ and $r_{recovery}\sim i.i.d. \beta\left( \omega_{recovery}, \kappa=20{,\alpha}_{recovery},\zeta_{recovery} \right)$.

**Clinical Characteristics**

For the purposes of this model, population outcomes of infection simulations are calibrated to empirical estimates of the clinical characteristics of TB patients in the pre-chemotherapy era [9],notably the case fatality ratio, disease duration, and proportion of cases resolved amongst symptomatic TB patients. To parameterize the disease burden for calibration, conceptual thresholds are imposed on the disease burden to define the onset/resolution of symptoms and the point of death. Such thresholds have been used in other within-host mathematical models as instruments for analyzing the transition from subclinical to detectable disease in ovarian cancer [10]. The mathematical “symptom threshold”, $\sigma$, is defined as the disease burden above which the patient is considered to experience severe enough symptoms for clinical presentation and diagnosis and below which the patient experiences a clinically inapparent infection and/or a resolution of symptoms. The mathematical “death threshold”, $\delta$, is defined as the disease burden above which the patient expires. For any individual infection, the duration of disease (specifically, the duration of symptomatic disease) can then be calculated as:

$$D(p)=\left[ \left( min(t|t\mathbb{\in N ,}N_{t}\geq\delta) \right)\vee\left( min(t|t\mathbb{\in N ,}N_{t-1}\geq\sigma, N_{t}<\sigma) \right) \right]-\left[ min(t|t\mathbb{\in N ,}N_{t}\geq\sigma) \right]$$

For a population $\mathbb{P\in}\left\{ 1,\cdots,P \right\}$, each member with a disease burden $N_{t}\left( p \right)$, the case-fatality ratio can be calculated as:

$$M\mathbb{(P)=}\frac{\left| \left\{ \mathbb{P} | max\left( N_{t}\left( p \right) \right)>\delta\right\} \right|}{\left| \left\{ \mathbb{P} | max\left( N_{t}\left( p \right) \right)>\sigma\right\} \right|}$$

and the proportion of cases which self-resolve can be calculated as:

$$R\mathbb{(P)=}\frac{\left| \left\{ \mathbb{P} | N_{t}\left( p \right)>\sigma,N_{t\mathbb{+N}}\left( p \right)\leq\sigma\right\} \right|}{\left| \left\{ \mathbb{P} | max\left( N_{t}\left( p \right) \right)>\sigma\right\} \right|}$$

**Parameterization and Sampling**

Ranges of values for each parameter that were deemed plausible on an a-priori basis are detailed in Table 1. The probability of transition from progression to recovery $\left( \Pr\left( S^{\left( t+1 \right)}=recovery | S^{\left( t \right)}=progression \right) \right)$ was defined by the limits of 0.1%-29.29% per week. The lower bound of this range, equivalent to a 5% probability that individuals transition in the first year of progression, was used considering that roughly 45% of patients survive the first five years of disease [9]; therefore, we speculated at least 10% of those survivors would begin to recover within the first year. The upper bound of this range, equivalent to a 75% probability that individuals transition in the first month of progression, was used considering that patients delay seeking treatment for an average of 25-32 days after symptom onset [11] and that clinical diagnostic criteria of pulmonary TB include a history of cough for 2-3 weeks [12,13]; therefore we speculated that at least one quarter of TB patients experience a continuous worsening of disease over the first month of symptoms. The probability of transition from recovery to progression $\left( \Pr\left( S^{\left( t+1 \right)}=progression | S^{\left( t \right)}=recovery \right) \right)$, analogous to the reactivation of latent TB infection, was defined by the limits of 0.001% to 0.00001% per week, equivalent to the benchmarks that minimum of 0.05% and a maximum of 5% of patients transition in the first year of recovery . These limits were defined with consideration that the yearly probability of reactivation latent TB infection has been estimated as 0.084% per year in asymptomatic individuals [14]. In light of these estimates, we assumed that the probability of symptomatic (recovering) patients experiencing symptom recrudescence would not be substantially less than the probability of reactivation from latency in immunocompetent adults but could be as high as two orders of magnitude greater.

The “death threshold” $(\delta)$ was allowed to vary from 10^7^ to 10^10^ units, using an analogous reference frame estimated from the maximum measured bacillary burdens of non-human primates suffering from asymptomatic and fulminant TB disease [15]. To ensure there is always a plausible separation between the “death threshold” and the “symptom threshold” ($\sigma$), the latter was defined by the log-difference $\log_{10} \left( \delta\right)-\log_{10} \left( \gamma\right)$, a “window width” parameter which defined the extent of disease progression between the onset of symptoms and death. The “window width” was allowed to vary between benchmarks of 10^4^ to 10^7^ units informed by the typical variations between bacillary burdens of asymptomatic/latent and symptomatic non-human primates. Therefore, deriving the “symptom threshold” from this log-difference allowed the “symptom threshold” to vary between 1 to 10^6^ units [15–18].

The bounds of the distribution of exponential rate constants in the progression phase (${[\alpha}_{progression},\zeta_{progression}]$) were defined according to the benchmarks that no patient in the progression phase would progress from asymptomatic/subclinical disease to death in less than 90 days – informed by the slow pathophysiology of clinical TB and early studies which found overall mortality in the first year of disease to be no greater than 30% [19]. We define the partition of “progression” and “recovery” phases according to the boundary of their exponential rate constants such that any patient who remains continuously in the progression phase would die in a maximum of 5 years of symptomatic disease. Therefore, for any patient $\frac{log\left( \frac{\delta}{\sigma} \right)}{90}\leq r_{progression}\leq\frac{log\left( \frac{\delta}{\sigma} \right)}{260\times7}$ and thus ${[\alpha}_{progression},\zeta_{progression}]=\left[ \frac{log\left( \frac{\delta}{\sigma} \right)}{90},\frac{log\left( \frac{\delta}{\sigma} \right)}{260\times7} \right]$. Assuming a reasonable value of $\frac{\delta}{\sigma}={10}^{4}$, the limits of the distribution of exponential rate constants in the progression phase were specified as 0.005 and 0.102 per day, equivalent to population doubling times in the of 137 days to 6 days, respectively.

The upper bound of the recovery phase exponential rate constant $\left( \zeta_{recovery} \right)$ was similarly defined according to the above definition such that any patient continuously progressing would die in a maximum of 5 years; consequentially, we define that, if any patient continuously in the recovery phase were to die, it would occur after no less than 5 years of symptomatic disease. The lower bound of the recovery phase exponential rate constant ($\alpha_{recovery}$) – the fastest allowable rate of spontaneous recovery – was informed by data on the average rate of recovery with the assistance of bactericidal therapies. It has been estimated that a patient receiving effective drug therapy will experience sputum conversion no sooner than an average of 35 days after initiation [20]. If one assumes that a symptomatic (spontaneously recovering) patient receiving no drug therapy will recover no faster than ¼ the rate of a patient receiving drug therapy, then for any patient $\frac{log\left( \frac{\sigma}{\delta} \right)}{35\times4}\leq r_{recovery}\leq\frac{log\left( \frac{\delta}{\sigma} \right)}{365\times5+1}$ and thus $\left[ \alpha_{recovery},\zeta_{recovery} \right]=\left[ \frac{log\left( \frac{\sigma}{\delta} \right)}{35\times4},\frac{log\left( \frac{\delta}{\sigma} \right)}{365\times5+1} \right]$. Assuming a reasonable value of $\frac{\delta}{\sigma}={10}^{4}$, the limits of the distribution of exponential rate constants in the recovery phase were specified as -0.066 and 0.005 per day, equivalent to a population half-life of 11 days and a population doubling time in the range of 137 days, respectively.

Modes of the population distributions of these exponential rate constants $\left( \omega_{progression},\omega_{recovery} \right)$ were allowed to vary across the full range of allowable values for each respective phase.

For each of the 2,000,000 simulations of the individual-level model, Latin hypercube sampling was used to draw one value from the specified ranges of each parameter to create 2,000,000 six-parameter sets. Transition probabilities from progression to recovery and vice versa were conceptualized as characteristics of the cohort (i.e., one value per cohort), with each individual patient trajectory comprised as stochastic realizations of these probabilities. Additionally, symptom and death thresholds were conceptualized as reference frames with which to compare individuals’ progression, and were therefore fixed for all patients (i.e., one value each per cohort). Parameter values for these transition probabilities and thresholds were uniformly sampled on log_10_-transformed scales. By contrast, rate constants of progression and recovery were conceptualized as distributional values – the mode was selected for a given cohort, and each individual’s personal rate of progression and recovery was drawn from a beta distribution around that mode. As the exponential rate constants ranged across both positive and negative values, the modal (cohort-level) values were sampled on the log-modulus-transformed scale [21], defined here as $T\left( r \right)=\frac{\left| r \right|}{r}\times log(100\times\left| r \right|+1)$.

**Importance Resampling**

Following initial Latin hypercube sampling [22], importance resampling [23] was used to generate Bayesian posteriors for each parameter as described below. A joint likelihood function was generated to describe probable values of the clinical characteristics of a cohort of TB cases from the pre-chemotherapy era [9]. The case-fatality ratio likelihood was modeled as a binomial distribution with p=0.55 and parameters $\left( k=\left| \left\{ \mathbb{P} | max\left( N_{t}\left( p \right) \right)>\delta\right\} \right| , n=\left| \left\{ \mathbb{P} | max\left( N_{t}\left( p \right) \right)>\sigma\right\} \right| \right)$ populated from values of each realized simulation. The likelihood for the median disease duration of a cohort was modeled as a symmetric beta distribution $\beta\left( \omega=0.5 , \kappa=8 \right)$ scaled to a range [1 , 5] years (modal equivalent of 3 years). This concentration value was used to define a broad likelihood function around the median duration (standard deviation of 8 months). The likelihood of the proportion of cases which self-resolve was modeled as a uniform distribution on the interval [0.1, 1.0]. Each parameter set was weighted according to the quotient of the set’s joint likelihood, L(*θ_i_* ; *x*), and the marginal likelihood of all sets:

$$q_{i}=\frac{L\left( \theta_{i};x \right)}{\sum_{j=1}^{n} L\left( \theta_{j};x \right)}$$

Each parameter value was then resampled from the Latin hypercube according to its weight to construct its posterior distribution.

**Diagnosis and Treatment**

To simulate the potential impact of diagnosis and treatment on the cumulative disease burden and time spent symptomatic among patients in a cohort, we compare various scenarios using the maximum likelihood parameter set identified above. Diagnosis and treatment is conceptualized as a weekly probability of detection, dependent upon disease burden. If a patient initiates treatment at time *t* (D_t_=1), she is removed from the infectious cohort before time *t*+1. The weekly probability of initiating treatment, Pr(D_t_=1 | N_t_), is set equal to zero at any disease burden below the symptom threshold. This probability increases logarithmically to a maximum p_max_ (defined as Pr(D_t_=1|N_t_=δ)) when the disease burden equals the death threshold such that:

$$\Pr\left( D_{t}=1|N_{t} \right)= \frac{\log_{10} N_{t}-\log_{10} \sigma}{\log_{10} \delta-\log_{10} \sigma}\times p_{max}$$

This disease-dependent probability of detection ensures that patients with nearly asymptomatic disease are unlikely to be diagnosed while patients near death are most likely to be diagnosed.

When the cohort is simulated with the introduction of diagnosis and treatment (each patient with a possibility of being detected and starting on treatment [Pr(D_t_(p)=1)] at each time *t*), the case detection proportion (commonly called the “case detection rate”, or CDR) of the entire cohort can be defined as the proportion of symptomatic patients detected prior to death or self-resolution:

$$CDR\mathbb{(P)=}\frac{\left| \left\{ \mathbb{P} | max(D_{t}\left( p \right))=1 \right\} \right|}{\left| \left\{ \mathbb{P} | max\left( N_{t}\left( p \right) \right)>\sigma\right\} \right|}$$

Values for p_max_ (weekly probability of initiating treatment at the time of maximum symptom burden) were sampled randomly from a uniform distribution on the log10 scale from 10-5 to 1.0 (median: 1.0% probability of detection per week), and one cohort of 1,000 patients each was simulated for each of the 1,000 p_max_ values sampled (holding all other parameters constant at their maximum likelihood values). The pseudo-likelihood associated with each value for p_max_ was calculated using the estimated 2015 global CDR of 59% [95% CI: 50-70%] [24]. The pseudo-likelihood function was parameterized as a normal distribution with a mean of 59% and a standard deviation of 5.1%.

The potential impact of treatment initiation was then estimated by comparing the cumulative burden-time of the cohort with and without detection and treatment. For each patient, the total burden-time is defined as the area under the curve of (disease burden above the symptom threshold) versus time (see Figure S4 for a graphical representation). If the time of symptom onset for a given patient *p* is a=[min(*t*|*t*∈N ,N_t_≥σ)] and the time of exit from the cohort (due to death, cure, detection, or end of follow-up) is b, then the patient’s total burden-time is:

$$\mathbb{B(}p)=\int_{a}^{b} N_{t} dt-\int_{a}^{b} \sigma dt$$

The cumulative burden-time for a population of *P* symptomatic patients can then be calculated as $\sum_{1}^{P} \mathbb{B(}p)$.

**SUPPLEMENTARY RESULTS**

Of the 2,000,000 simulated patient cohorts (each containing 1,000 patients), 76,544 (3.8%) had no TB patients who progressed to symptomatic disease within 5 years of infection. In an additional 34,596 cohorts (1.7%), all symptomatic TB cases suffered symptoms for at least five years before death or resolution; these cohorts were excluded from further analysis. Of the remaining cohorts, the epidemiological characteristics of TB were highly varied with case fatality ratios ranging from 0%-100% [Interquartile range (IQR): 7.5%, 80%] and median durations ranging from 1 week to 5 years [IQR:0.48 years, 1.3 years]. After applying the joint likelihood function to differentially weight plausibly realistic cohorts from discernibly unrealistic cohorts, 551,100 cohorts (28% of the total) had results consistent with historical estimates of the natural history of TB (joint likelihoods greater than zero). Amongst these plausible cohorts, the median case fatality ratio was 42.5% [IQR: 18%, 67%] and the median duration of disease for the 50^th^ percentile of cohorts was 1.5 years [IQR:1.2 years, 1.9 years]; however, amongst those cohorts accounting for 90% of total likelihood mass (n=20,770), the median duration of disease was substantially longer at 2.3 years [IQR:1.9 years, 2.7 years]. Notably, these results indicate a closer recapitulation of historical case fatality ratio estimates (target: 55%; posterior IQR: 54-56%) than disease duration estimates (target: 3.0 years; posterior IQR: 2.1-2.8 years). This suggests that, in the simplified modeling approach used here, cohorts with case fatality proportions that are most similar to the reported values (i.e., highest likelihood cohorts) have mean disease durations that are somewhat shorter than those reported. In other words, a disease process that results in 55% case fatality in our simplified model generally progresses more rapidly than would be expected if the mean disease duration were actually 3.0 years. Further work is justified to discern whether this discrepancy is more likely a reflection of the simplified modeling framework utilized here (e.g., if addition of further model complexity would result in less of a discrepancy) or whether this apparent discrepancy may suggest actual measurement error in pre-chemotherapy studies that relied largely on reported symptoms for their estimates of disease duration.

Of the 1,000 cohorts simulated with detection and treatment initiation, 6.2% demonstrated case detection proportions consistent with global estimates (median: 59%; 95% CI: 50-70%). After importance resampling, the median posterior CDR in these simulated cohorts was 57.2% (95% UR: 47.9-69.8%). In the absence of detection and treatment, mortality using the maximum likelihood parameter set identified above was 53.2%. When the maximum likelihood estimate of the weekly detection probability (1.4% per week) was applied, mortality in this cohort dropped to 17.2% (omitting possible deaths during treatment). Although mortality was not calibrated to data from patients receiving treatment, this estimate was nevertheless very similar to the reported 2015 global mortality rate of 17.3% [24].

Without diagnosis and treatment initiation, the cohort’s cumulative burden-time was 11.0 log(unit-weeks). When the maximum likelihood probability of detection and treatment initiation was applied (from above), the burden-time was reduced to 10.5 log(unit-weeks), a 4.5% reduction in cumulative burden time (on the log_10_ scale). We then explored the potential impact of improved diagnosis using Xpert® MTB/RIF over sputum microscopy (assuming that the probability of diagnosis and treatment is proportional to the sensitivity of the assay used). When the probability of weekly detection was increased 1.4-fold (to represent the improved sensitivity of Xpert® MTB/RIF over sputum microscopy [25,26]), the log burden-time was further reduced to 10.3 log(unit-weeks). This reduction represents a 6.3% reduction in cumulative burden-time compared with no detection and treatment but only a 1.9% reduction in burden-time compared with detection and treatment based on the existing standard of care. To the extent that cumulative burden-time also reflects cumulative transmission potential (especially if expressed on the log scale in our model), these results may illustrate why improvements in detection and treatment can yield important reductions in mortality without dramatically impacting incidence – as has been seen both with the scale-up of DOTS in the 1990s and more recently projected for the scale-up of Xpert® MTB/RIF over the past five years [27,28].**REFERENCES**

1. Waitt CJ, Squire SB. A systematic review of risk factors for death in adults during and after tuberculosis treatment. Int J Tuberc Lung Dis. **2011**; 15(7):871–885.

2. Lopez B, Aguilar A, Orozco H, Burger M, Espitias C, Ritacco V, et al. A marked difference in pathogenesis and immune response induced by different *Mycobacterium tuberculosis* genotypes. Clin Exp Immunol. **2003**; 133:30–37.

3. Dormans J, Burger M, Aguilar D, Hernandez-Pando R, Kremer R, Roholl P, et al. Correlation of virulence , lung pathology , bacterial load and delayed type hypersensitivity responses after infection with different *Mycobacterium tuberculosis* genotypes in a BALB/c mouse model. Clin Exp Immunol. **2004**; 137:460–468.

4. Marquina-Castillo B, Garcia-Garcia L, Ponce-de-Leon P, Jimenez-Corona M, Bobadilla-del-Valle M, Cano-Arellano B, et al. Virulence , immunopathology and transmissibility of selected strains of *Mycobacterium tuberculosis* in a murine model. Immunology. **2009**; 128(1):123–133.

5. Gill WP, Harik NS, Whiddon MR, Liao RP, Mittler JE, Sherman DR. A replication clock for Mycobacterium tuberculosis. Nat Med. **2009**; 15(2):211–214.

6. McDaniel MM, Krishna N, Handagama WG, Eda S, Ganusov V. Quantifying limits on replication, death, and quiescence of *Mycobacterium tuberculosis* in mice. Front Microbiol. **2016**; 7:862.

7. Salie M, Merwe L van der, Möller M, Daya M, Spuy GD van der, Helden PD Van, et al. Associations between human leukocyte antigen class i variants and the *Mycobacterium tuberculosis* subtypes causing disease. J Infect Dis. **2014**; 209:216–223.

8. Caws M, Thwaites G, Dunstan S, Hawn TR, Lan N, Thuong N, et al. The influence of host and bacterial genotype on the development of disseminated disease with *Mycobacterium tuberculosis*. PLoS Pathog. **2008**; 4(3):e1000034.

9. Tiemersma EW, Werf MJ van der, Borgdorff MW, Williams BG, Nagelkerke NJD. Natural history of tuberculosis: duration and fatality of untreated pulmonary tuberculosis in HIV negative patients: A systematic review. PLoS One. **2011**; 6(4):e17601.

10. Botesteanu D, Lee J, Levy D. Modeling the dynamics of high-grade serous ovarian cancer progression for transvaginal ultrasound-based screening and early detection. PLoS One. **2016**; 11(6):e0156661.

11. Sreeramareddy CT, Panduru K V, Menten J, Ende J Van den. Time delays in diagnosis of pulmonary tuberculosis: a systematic review of literature. BMC Infect Dis. **2009**; 9:91.

12. The Centers for Disease Control and Prevention (CDC). Core curriculum on tuberculosis : what the clinician should know. 6th Ed. Centers Dis. Control Prev. Natl. Cent. HIV/AIDS, Viral Hepatitis, STD, TB Prev. Div. Tuberc. Elimin. Atlanta; 2013.

13. World Health Organization (WHO), International Union Against Tuberculosis and Lung Disease (IUATLD), Royal Netherlands Tuberculosis Association (KNCV). Revised international definitions in tuberculosis control. Int J Tuberc Lung Dis. **2001**; 5(3):213–215.

14. Shea KM, Kammerer JS, Winston CA, Navin TR, Horsburgh CR. Estimated rate of reactivation of latent tuberculosis infection in the United States, overall and by population subgroup. Am J Epidemiol. **2014**; 179(2):216–225.

15. Lin PL, Rodgers M, Smith L, Bigbee M, Myers A, Bigbee C, et al. Quantitative comparison of active and latent tuberculosis in the cynomolgus macaque model. Infect Immun. **2009**; 77(10):4631–4642.

16. Capuano SV 3rd, Croix DA, Pawar S, Zinovik A, Myers A, Lin PL, et al. Experimental *Mycobacterium tuberculosis* infection of cynomolgus macaques closely resembles the various manifestations of human M. tuberculosis infection. Infect Immun. **2003**; 71(10):5831–5844.

17. Lin PL, Coleman T, Carney JPJ, Lopresti BJ, Tomko J, Fillmore D, et al. Radiologic responses in cynomolgus macaques for assessing tuberculosis chemotherapy regimens. Antimicrob Agents Chemother. **2013**; 57(9):4237–4244.

18. Lin PL, Ford CB, Coleman MT, Myers AJ, Gawande R, Ioerger T, et al. Sterilization of granulomas is common in active and latent tuberculosis despite within-host variability in bacterial killing. Nat Med. Nature Publishing Group; **2014**; 20(1):75–79.

19. Berg A. The prognosis of open pulmonary tuberculosis. A clinical-statistical study. Acta Tuberc Scand. **1939**; Supplement:1–206.

20. Kanda R, Nagao T, Tho N Van, Ogawa E, Murakami Y, Osawa M, et al. Factors affecting time to sputum culture conversion in adults with pulmonary tuberculosis: a historical cohort study without censored cases. PLoS One. **2015**; 10(11):e0142607.

21. John J, Draper N. An alternative family of transformations. Appl Stat. **1980**; 29(2):190–197.

22. Stein M. Large sample properties of simulations using Latin hypercube sampling. Technometrics. **1987**; 29(2):143–151.

23. Smith AFM, Gelfand AE. Bayesian statistics without tears: a sampling-resampling perspective. Am Stat. **1992**; 46(2):84–88.

24. World Health Organization (WHO). Global tuberculosis report 2016. Geneva; 2016.

25. Davis JL, Cattamanchi A, Cuevas LE, Hopewell PC, Steingart KR. Diagnostic accuracy of same-day microscopy versus standard microscopy for pulmonary tuberculosis: a systematic review and meta-analysis. Lancet Infect Dis. Elsevier Ltd; **2013**; 13(2):147–154.

26. Steingart KR, Schiller I, Horne DJ, Pai M, Boehme CC, Dendukuri N. Xpert ® MTB / RIF assay for pulmonary tuberculosis and rifampicin resistance in adults. Cochrane Database Syst Rev. **2014**; Jan 21(1):CD009593.

27. Dowdy DW, Chaisson RE. The persistence of tuberculosis in the age of DOTS: reassessing the effect of case detection. Bull World Health Organ. **2009**; 87(4):296–304.

28. Menzies NA, Cohen T, Lin HH, Murray M, Salomon JA. Population health impact and cost-effectiveness of tuberculosis diagnosis with Xpert MTB/RIF: a dynamic simulation and economic evaluation. PLoS Med. **2012**; 9(11):e1001347.

**SUPPLEMENTARY FIGURES**

**Figure S1. Values of the Symptom Window Width Consistent with Observed Cohort Data**.

The symptom width window was used to define the symptom threshold by determining the difference between the death threshold ($\delta)$ and the symptom threshold (σ). Model input values (prior distributions) were sampled uniformly on the log-transformed scale between reasonable bounds selected on an a priori basis (denoted by vertical dashed lines). Histogram densities show the proportion of 2 million weighted parameter values that were most consistent with observed cohort data from the pre-chemotherapy era (posterior distributions).

**Figure S2.** **Values of Threshold Parameters Consistent with Observed Cohort Data.**

The symptom threshold parameter was derived as the difference between the log-transformed death threshold and symptom window width. Therefore, the model input values (prior distribution) for the symptom threshold parameter was not uniformly sampled, but was derived from two uniformly sampled parameters (the death threshold and the symptom window width). Input values for the symptom threshold are depicted in darker shades behind the weighted values most consistent with observed data (depicted in lighter transparent shades). Input values for the death threshold were sampled uniformly on the log-transformed scale between the displayed bounds, and weighted values most consistent with observed cohort are depicted in green.

**Figure S3.** **Values of the Probability of Transition to Progression Consistent with Observed Cohort Data.**

Model input values (prior distributions) were sampled uniformly on the log-transfomred scale between the defined bounds (depicted as vertical dashed lines). Histogram densities depict the proportion of 2 million weighted parameter values most consistent with observed cohort data from the pre-chemotherapy era (posterior distribution). Probabilities of transition from the recovery phase to the progression phase are depicted on weekly and annualized scales.

**Figure S4. Evaluating the Impact of Detection and Treatment on TB Morbidity**

The impact of detection and treatment was evaluated by comparing a cohort’s cumulative burden-time in the absence or presence of an intervention. (A) A representative cohort in which no patients are detected and treated, as described in the Methods and Figure 2. Emphasized are the disease trajectories of three patients (labeled 1-3). (B) The results of the same cohort when each patient has a weekly probability of detection and treatment, dependent on his or her disease burden in each week. Patients who are detected and treated before surpassing the death threshold (or self-resolution or the end of follow-up) are depicted in violet, with the times of detection/treatment overlaid as purple points. (C) Comparison of the trajectories of Patients 1-3 in the absence and presence of detection and treatment. Patient 1 does not receive treatment before surpassing the death threshold. Patients 2-3 are detected and treated and are removed from the infectious cohort at the time of detection and treatment. (D) Cumulative burden-time is calculated as the sum of the areas under each patient’s disease burden curve (above the symptom threshold). Shaded areas (of any color) denote this cumulative burden-time for each patient in the absence of detection and treatment. Red shaded areas represent each patient’s burden-time prior to the point of detection and treatment. Green shaded areas represent the burden-time averted through detection and treatment.
